# Structure Evolution and Multiferroic Properties in Cobalt Doped Bi_4_NdTi_3_Fe_1-*x*_Co_*x*_O_15_-Bi_3_NdTi_2_Fe_1-*x*_Co_*x*_O_12-*δ*_ Intergrowth Aurivillius Compounds

**DOI:** 10.1038/srep43540

**Published:** 2017-03-08

**Authors:** D. L. Zhang, W. C. Huang, Z. W. Chen, W. B. Zhao, L. Feng, M. Li, Y. W. Yin, S. N. Dong, X. G. Li

**Affiliations:** 1 Hefei National Laboratory for Physical Sciences at the Microscale, Department of Physics, University of Science and Technology of China, Hefei 230026, China; 2 Department of Physics and Astronomy, University of Nebraska, Lincoln, NE 68588, USA; 3 Key Laboratory of Materials Physics, Institute of Solid State Physics, CAS, Hefei 230026, China; 4 Collaborative Innovation Center of Advanced Microstructures, Nanjing 210093, China

## Abstract

Here, we report the structure evolution, magnetic and ferroelectric properties in Co-doped 4- and 3-layered intergrowth Aurivillius compounds Bi_4_NdTi_3_Fe_1-*x*_Co_*x*_O_15_-Bi_3_NdTi_2_Fe_1-*x*_Co_*x*_O_12-*δ*_. The compounds suffer a structure evolution from the parent 4-layered phase (Bi_4_NdTi_3_FeO_15_) to 3-layered phase (Bi_3_NdTi_2_CoO_12-*δ*_) with increasing cobalt doping level from 0 to 1. Meanwhile the remanent magnetization and polarization show opposite variation tendencies against the doping level, and the sample with *x* = 0.3 has the largest remanent magnetization and the smallest polarization. It is believed that the Co concentration dependent magnetic properties are related to the population of the Fe^3+^ -O-Co^3+^ bonds, while the suppressed ferroelectric polarization is due to the enhanced leakage current caused by the increasing Co concentration. Furthermore, the samples (*x* = 0.1–0.7) with ferromagnetism show magnetoelectric coupling effects at room temperature. The results indicate that it is an effective method to create new multiferroic materials through modifying natural superlattices.

The search for multiferroic materials combining electric and magnetic properties in a single phase has attracted a lot of attention in the perspective of future spintronic or magnetoelectronic devices^1^,^2^,^3^. Unfortunately, only a handful of single phase multiferroics have been discovered so far, and most of them are not suitable for practical applications at present, either because the room temperature polarization/magnetization is too small or their mutual coupling is too weak^4^,^5^,^6^. Therefore people are still hunting for new multiferroic systems^2^,^7^. One possible route for designing single-phase multiferroic materials is to start from a series of well-established ferroelectrics and create additional functionality by incorporating magnetic ions into these systems^1^,^8^,^9^. A promising class of materials for this purpose is the so-called Aurivillius phases with a naturally layered perovskite-related crystal structure, which consists of *n* perovskite-like layers (A_*n*−1_B_*n*_O_3*n*+1_)^2−^, stacked along the [001] direction, and separated by fluorite-like (Bi_2_O_2_)^2+^ layers^10^. The overall chemical composition is thus Bi_2_A_*n*−1_B_*n*_O_3*n*+3_, where many different cations can be incorporated on the A and B sites within the perovskite-like layers^11^. The series of Aurivillius phase compounds are well known for their excellent ferroelectric properties with very low fatigue^12^, and offer great potential for tailoring specific properties by varying different ionic compositions or even number of layers^8^,^13^,^14^. By doping with magnetic cations, the bismuth-based Aurivillius phase compounds were found to have a room temperature ferromagnetic order besides the natural ferroelectricity, indicating their multiferroic potential^8^,^9^,^15^,^16^,^17^. Moreover, the Sm and Co co-doped 3-layered (*n* = 3) Bi_4-*x*_Sm_*x*_Ti_3-*x*_Co_*x*_O_12-δ_ (0 ≤ *x* ≤ 0.07) ceramics show a magnetoelectric (ME) coupling coefficient of 0.65 mV/cm∙Oe at room temperature^15^, while the 5-layered (*n* = 5) SrBi_5_Fe_0.5_Co_0.5_Ti_5_O_18_ ceramic was found to show a ME coupling coefficient of 0.27 mV/cm∙Oe^8^. Thus this kind of material is one of the excellent choices for ME applications and deserves further investigation.

Furthermore, due to the complexity of the structure, an interesting phenomenon called intergrowth has been reported in Aurivillius compounds^18^,^19^,^20^. It has been demonstrated that the ferroelectricity of 4- and 3-layered intergrowth compounds is larger than that of individual 4- or 3-layered compound^21^,^22^. However, the effects of magnetic ions doping on ferroelectric, magnetic and multiferroic properties are still unknown in such an intergrowth superlattice system.

Here we successfully synthesized a series of Bi_4_NdTi_3_Fe_1-*x*_Co_*x*_O_15_-Bi_3_NdTi_2_Fe_1-x_Co_x_O_12-δ_ (BNTFC-*x*) compounds with different ratios of 4- and 3-layered Aurivillius intergrowth superlattice structure. It was found that as the cobalt doping concentration increases from 0 to 1, the samples suffer a structure evolution from 4-layers to 3-layers. The observed magnetic and ferroelectric properties can be well explained by the magnetic ions doping and the intergrowth structure evolution.

## Results and Discussion

**Figure 1:**
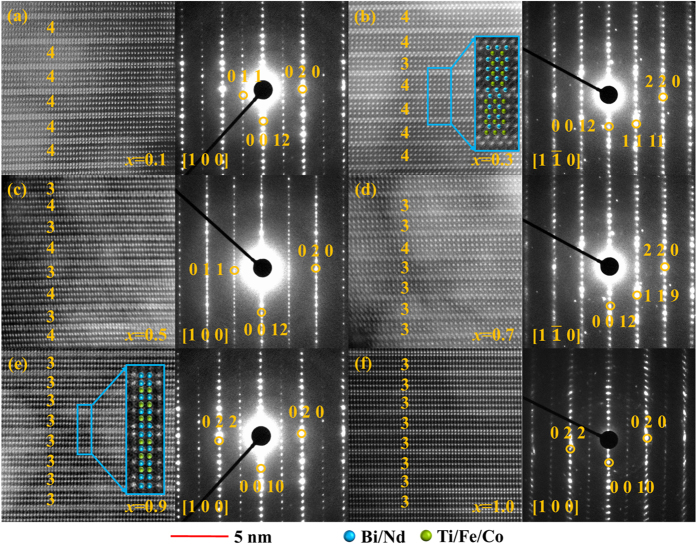
HAADF images and SAED patterns of BNTFC–*x*: (**a**) *x* = 0.1; (**b**) *x* = 0.3; (**c**) *x* = 0.5; (**d**) *x* = 0.7; (**e**) *x* = 0.9; (**f**) *x* = 1.0. The big white spots represent the Bi (Nd) atoms, while the small spots located near the center of Bi/Nd lattice represent the Ti/Fe/Co atoms. The inset in (**b**) and (**e**) is a magnifying image for 4- and 3-layered phase, respectively, where the azury and green sphere denote Bi/Nd and Ti/Fe/Co atoms, respectively. For (**a**,**c**,**e** and **f**), the viewing direction is along [100], while for (**b**) and (**c**) the viewing direction is along 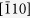. For *x* ≤ 0.5, the marked diffraction spots belong to 4-layered phase, while for *x* > 0.5 the marked diffraction spots belong to 3-layered phase.

To clarify the structure evolution of the Bi_4_NdTi_3_Fe_1-*x*_Co_*x*_O_15_-Bi_3_NdTi_2_Fe_1-*x*_Co_*x*_O_12-*δ*_ (BNTFC-*x*) compounds, the high-angle annular dark-field (HAADF) images and selected area electron diffraction (SAED) were performed, as shown in Fig. 1. In the HAADF images, the big bright spots stand for the location of the Bi/Nd atoms while the small spots located near the center of Bi/Nd lattice represent the Ti/Fe/Co atoms as depicted in the inset of Fig. 1(b) (4-layered) and (e) (3-layered). It can be seen that the fluorite-like (Bi_2_O_2_)^2+^ layers and peroskite-like (A_*n*−1_B_*n*_O_3*n*+1_)^2−^ layers are stacking along *c* direction, indicating that all compounds have a typical Aurivillius layered structure. From the SAED patterns, the electron incidence direction, namely the view direction, can be obtained, as marked in Fig. 1. As shown in Fig. 1(a), the 4-layered lattice can be clearly distinguished for *x* = 0.1. As the Co concentration increases to 0.3, the 3-layered lattice begins to appear as shown in Fig. 1(b). While *x* increases to 0.5, the 4-layered and 3-layered structures alternately stack along the [001] direction (shown in Fig. 1(c)). With the *x* further increasing to 0.7, the 3-layered structure starts to dominate (shown in Fig. 1(d)), and at last for *x* = 0.9 and 1 components, the 4-layered Aurivillius structure almost disappears (shown in Fig. 1(e) and (f)). The HAADF results indicate that increasing Co doping level makes the BNTFC-*x* compound experiencing a structure evolution from 4-layered structure to 3-layered structure. This intergrowth phenomenon has also been reported in Sr_*x*_Bi_7-*x*_Fe_1.5_Co_1.5_Ti_3_O_21-*δ*_, which undergoes a phase evolution from 6-layers to 4-layers when the concentration of A-site doped strontium increases from 0 to 1^14^.

**Figure 2:**
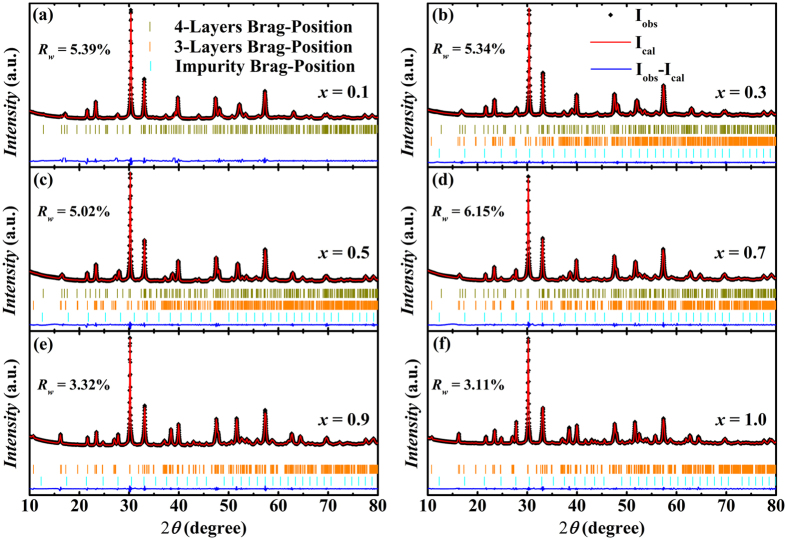
XRD patterns and refined results of BNTFC-*x* samples with (**a**) *x* = 0.1; (**b**) *x* = 0.3; (**c**) *x* = 0.5; (**d**) *x* = 0.7; (**e**) *x* = 0.9; (**f**) *x* = 1.0.

**Figure 3:**
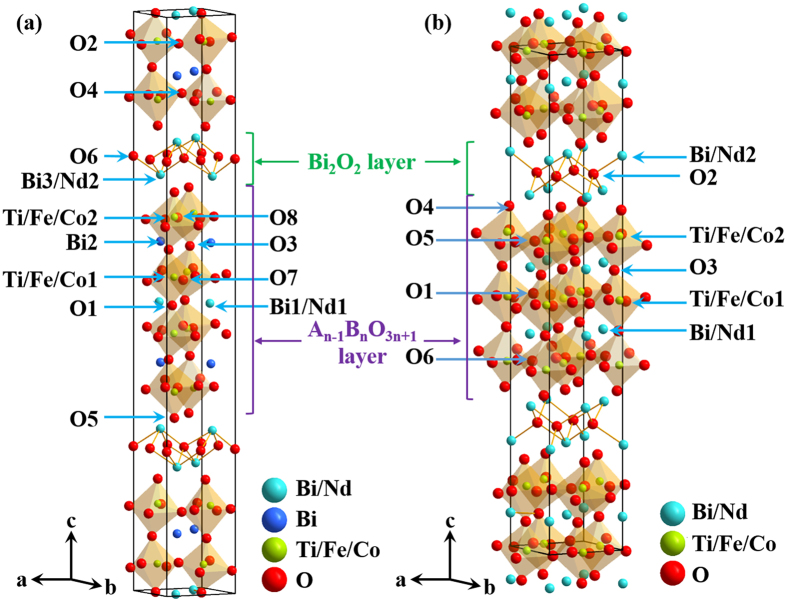
Schematic structure diagram of (**a**) 4- and (**b**) 3-layered phases, with atom positions marked in the figure.

The room temperature powder X-ray diffraction (XRD) patterns with data refined by Rietveld Method *via* Materials Analysis Using Diffraction (MAUD) program^23^,^24^ for BNTFC-*x* ceramics are shown in Fig. 2. Quantitative analysis confirms that three phases, including 4-layered phase Bi_4_NdTi_3_Fe_1-*x*_Co_*x*_O_15_ (*n* = 4), 3-layered phase Bi_3_NdTi_2_Fe_1-*x*_Co_*x*_O_12-*δ*_ (*n* = 3), and an impurity phase Bi_12_TiO_20_ coexist in the BNTFC-*x* system. The refinements were based on space group A2_1_am (No. 36) for 4-layered phase^25^, B2cb (No. 41) for 3-layered phase^26^ and I23 (No. 197) for Bi_12_TiO_20_ phase^27^. The good matching between experimental and calculated XRD patterns is demonstrated by the low *R*_*w*_ value for all compounds (*R*_*w*_ ≤ 6.15%), as shown in Fig. 2. To better understand and analysis the structures, the schematic structure diagrams of 4- and 3-layered phases are shown in Fig. 3(a) and (b), respectively. The Ti, Fe and Co cations were set to occupy the same position (B site in the center of each perovskite structure in the perovskite-like layer) with fixed Ti occupancy (3/4 in 4-layer phase, and 2/3 in 3-layer phase) and changeable occupancies of Fe and Co with the cobalt nominal doping level ((1-*x*)/4 for Fe and *x*/4 for Co in 4-layer phase, (1-*x*)/3 for Fe and *x*/3 for Co in 3-layer phase).

**Figure 4:**
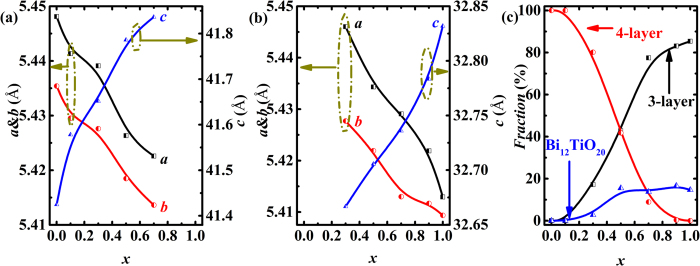
Lattice parameters of (**a**) 4-layered phase and (**b**) 3-layered phase; (**c**) the volume fraction of 4-, 3-layered and impurity (Bi_12_TiO_20_) phases.

The lattice parameters *a* and *b* obtained by refinement gradually decrease and *c* increases in both 4-layered (Fig. 4(a)) and 3-layered (Fig. 4(b)) phase with increasing cobalt concentration. As shown in Fig. 4(c), the volume fraction of 4- (3-) layered phase gradually decreases (increases) from 100% (0) to 0 (85.34%) as *x* increases from 0 to 1, also confirming that the samples suffer a structure evolution from 4-layered phase to 3-layered phase. The fraction of the Bi_12_TiO_20_ phase increases with the Co concentration in the samples with *x* < 0.5, and keeps at about 15% for *x* ≥ 0.5. The impurity phase (Bi_12_TiO_20_) is induced by the element loss during the structure evolution process, similar to the situation in Sr_*x*_Bi_7-*x*_Fe_1.5_Co_1.5_Ti_3_O_21-*δ*_ system^14^. According to the results of XRD patterns and HAADF images, the BNTFC-*x* compounds undergo a structure evolution with a two-phase modulated superlattice when the concentration of Co changes. In other words, the micro superlattice structure can be naturally controlled by the cobalt doping level.

In addition, the Fe/Co ions can occupy two non-equivalent positions (center of inner and outer octahedrons between two (Bi_2_O_2_)^2+^ layers) in 4- and 3-layered structures. In 4-layered Bi_5_Ti_3_FeO_15_, these octahedrally coordinated center sites are shared between Ti^4+^ and Fe^3+^ cations, and a quasi-random cation distribution is observed in experiments^25^,^28^. Similarly, it can be assumed that Ti^4+^, Fe^3+^, and Co^3+^ cations are randomly distributed in the octahedral center positions in both 4- and 3-layered structures. In perovskite-like compounds the most probable valence states of Fe and Co are + 3. According to Goodenough-Kanamori rules^9^,^29^,^30^,^31^, Fe^3+^ -O-Fe^3+^ and Co^3+^ -O-Co^3+^ superexchange interactions with the nature of antiferromagnetism exist in *x* = 0 and 1 samples, respectively, making the ground state of these two compounds to be antiferromagnetic. Based on the discussions on the occupations and interactions of Fe/Co cations, it can be expected that the structure evolution of BNTFC-*x* will have a significant impact on the physical properties, *e.g.* magnetism and ferroelectricity. To verify this, we systemically measured the magnetic and ferroelectric properties of BNTFC-*x* compounds, and discussed the relationship between the structure and properties as follows.

**Figure 5:**
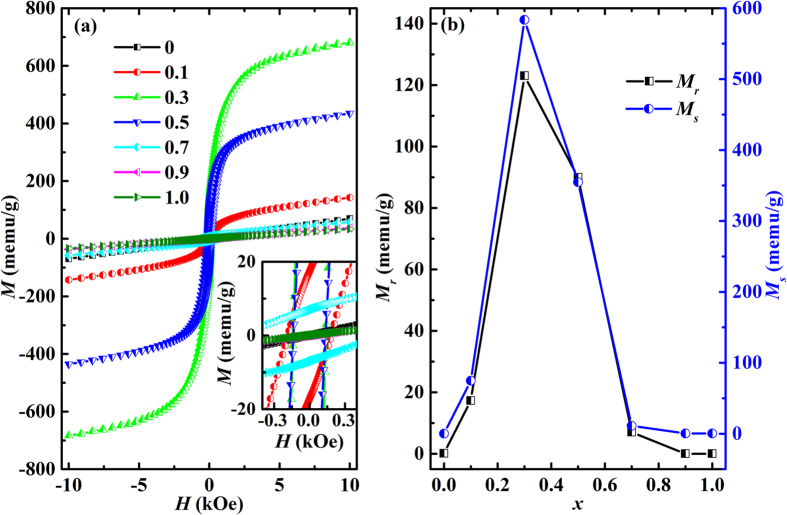
(**a**) Room temperature *M-H* hysteresis loops. Inset is the zoom of the main plot; (**b**) *M*_*r*_ and *M*_*s*_ as a function of cobalt doping level *x* for BNTFC-*x (x* = 0, 0.1, 0.3, 0.5, 0.7, 0.9 and 1).

The room temperature magnetic hysteresis loops for BNTFC-*x* are shown in Fig. 5(a). The magnetization (*M) versus* applied field (*H*) curves of *x* = 0.1 to 0.7 samples show a typical ferromagnetic hysteresis feature, while those for *x* = 0, 0.9 and 1.0 show a linear behavior. The remanent magnetization (*M*_*r*_) and saturation magnetization (*M*_*s*_, obtained from the *M-H* curves after deducting the linear part) gradually increase and reach a maximum with increasing Co content to *x* = 0.3, and then decreases with further increasing Co content, as shown in Fig. 5(b). It is known that the ground state of Bi_4_NdTi_3_FeO_15_ (*x* = 0) is antiferromagnetic. When Co^3+^ replaces parts of Fe^3+^ cations, some Fe^3+^ -O-Fe^3+^ chains will be destroyed, while the antiparallel Fe^3+^ and Co^3+^ superexchange interaction appears. Considering the difference of magnetic moments between a single Fe^3+^ (5.916 μ_B_) and a single Co^3+^ (4.899 μ_B_)^32^, the interaction of Fe^3+^ -O-Co^3+^ should contribute to a net magnetization, as discussed in Bi_5_Fe_0.5_Co_0.5_Ti_3_O_15_^33^ and Bi_4_NdFe_0.5_Co_0.5_Ti_3_O_15_^16^ ceramics. In the situation of *x* = 0.1, namely 10% Fe^3+^ ions are substituted by Co^3+^, the formation of Fe^3+^ -O-Co^3+^ bonds results in the appearance of ferromagnetism. In *x* = 0.3, more Fe^3+^ -O-Co^3+^ bonds form, leading to a larger remanent magnetization. While for *x* > 0.5, the decrease of the amount of Fe^3+^ -O-Co^3+^ bonds may lead to the diminution of magnetization. As for *x* = 0.9, the concentration of Fe^3+^ ions is too low to construct effective Fe^3+^ -O-Co^3+^ order, the system may be antiferromagnetic or paramagnetic at room temperature, just like Bi_5_Ti_3_FeO_15_^34^. Thus, it can be proposed that the magnetization of BNTFC-*x* may be mainly contributed by the population of Fe^3+^ -O-Co^3+^ local structure due to the cobalt doping^33^. Besides, the Ti/Fe/Co-O-Ti/Fe/Co angles may be affected by Co substitution^35^ and structure evolution^22^, which could also affect the observed magnetic properties. It should be noted that the most prominent remanent magnetization (*M*_*r*_ = 123 memu/g) at *x* = 0.3 is about 31 times larger than that of Bi_5_Ti_3_Fe_0.5_Co_0.5_O_15_ (3.9 memu/g)^33^, and comparable with that of Bi_4_NdTi_3_Fe_0.5_Co_0.5_O_15_ (165 memu/g)^16^ and Bi_4_NdTi_3_Fe_0.7_Ni_0.3_O_15_ (194 memu/g)^36^.

It has been reported that the doping of an Aurivillius phase with cobalt will lead to the generation of magnetic second-phase inclusions (Co/Fe-rich spinel phases) which volume fraction is too small to be visible in XRD but may be already enough to contribute significant ferromagnetic signal^9^,^37^,^38^. Generally, the Fe and Co-rich magnetic inclusions have a chemical formula Fe_3-*y*_Co_*y*_O_4_ (0 ≤ *y* ≤ 3), and the remanent magnetizations (0–20 emu/g) at room temperature decrease with increasing Co content^39^,^40^,^41^,^42^,^43^,^44^. Following the effective statistical method proposed by M. Schmidt *et al*.^38^, the volume fraction of the possible inclusions and their upper limit impact on magnetic contributions (*M*_*i*_*/M*_*r*_, where *M*_*i*_ is the remanent magnetization of the inclusions and *M*_*r*_ is that of the specimen) for the worst case scenario were carefully estimated *via* energy selective backscatter (ESB) image and energy dispersive X-ray analysis (EDX). For the samples with ferromagnetic signals, namely, *x* = 0.1, 0.3, 0.5, and 0.7, the magnetic contributions to the corresponding specimens of the inclusions are conservatively estimated to be about or smaller than 3.9%, 1.9%, 3.8%, and 1.5%, respectively. While for *x* = 0.9 and 1.0, the inclusions are paramagnetic at 300 K^44^,^45^, and have no magnetic contributions to the main phase. Based on the criteria of the comprehensive framework raised by M. Schmidt *et al*.^38^, we believe that the magnetic results do reflect the intrinsic ferromagnetic properties of the main phase. Detailed calculations are presented in Part I of the Supplementary Material.

**Figure 6:**
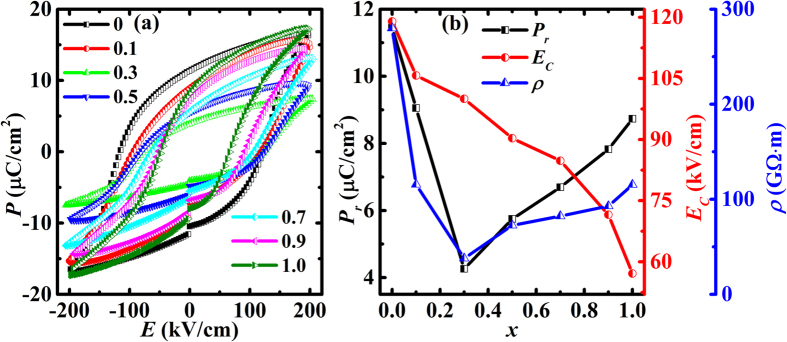
(**a**) Room temperature *P-E* hysteresis loops measured under standard bipolar mode (**b**) *P*_*r*_, *E*_*c*_, and *ρ vs. x* curves for BNTFC-*x*.

Figure 6 shows the room temperature polarization (*P) versus* electric field (*E*) curves of BNTFC-*x* samples, indicating that all specimen have a good ferroelectricity. With increasing the cobalt doping concentration, the remanent polarization *P*_r_ first decreases and reaches the minimum (4.26 μC/cm^2^) at *x* = 0.3, and then gradually increases with further doping. The obtained *P*_r_ for BNTFC-*x* is better than Bi_4_NdTi_3_Fe_0.5_Co_0.5_O_15_ (1 μC/cm^2^)^16^ as well as Bi_4_NdTi_3_Fe_0.7_Ni_0.3_O_15_ (4.3 μC/cm^2^)^36^, and comparable to that of Bi_5_Ti_3_Fe_0.5_Co_0.5_O_15_ ceramics (6.5 μC/cm^2^)^33^. While the coercive field *E*_c_ of BNTFC-*x* keeps decreasing as the cobalt concentration increases, which should be related to the smaller coercive field in 3-layered phase than 4-layered phase^21^. It should be noted that the *P*_*r*_*-x* curve (Fig. 6(b), black line) shows an opposite variation tendency as compared with *M*_*r*_*-x* curve (Fig. 5(b), black line), indicating a correlation between ferroelectricity and magnetism. For example, the minimum *P*_r_ and maximum *M*_*r*_ are observed in *x* = 0.3 sample. The *P*_*r*_*-x* curve indicates that the *P*_*r*_ values of the 4- and 3- layered intergrowth compounds (0.1 < *x* < 0.9) are smaller than that of individual 4- (*x* = 0) or 3- (*x* = 1) layered compound, which is opposite to previous reports (no magnetic ions doped in the samples)^21^,^22^, probably due to the magnetic ion doping in our system. Figure 6(b) (red line) shows the resistivity (*ρ*) for the BNTFC-*x* at room temperature. The similar variation tendency of *P*_*r*_ and *ρ* implies that the weakened ferroelectricity should be attributed to the increasing leakage current. Usually, magnetic ion doping will reduce the resistivity of a dielectric material and weaken the ferroelectric performance due to the strengthening of exchange interaction between magnetic ions^46^,^47^. This behavior is consistent with other multiferroic system, such as Co-doped BiFeO_3_^46^ and Fe-doped BaTiO_3_^47^.

**Figure 7:**
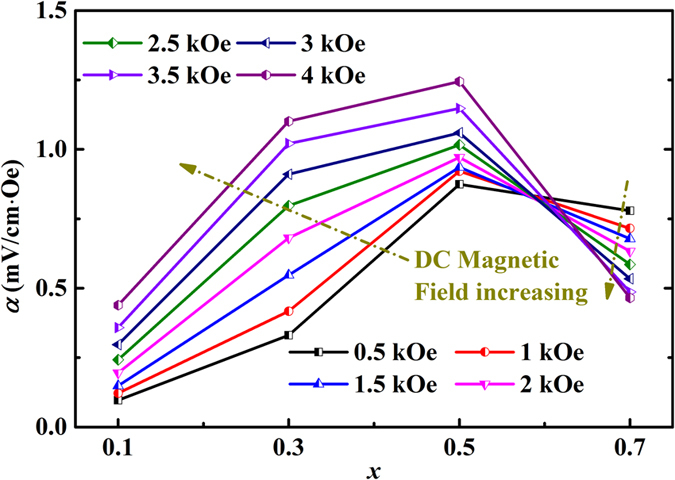
ME coefficients of BNTFC-*x* as a function of cobalt doping level *x*.

The magnetoelectric (ME) effects of the samples with ferromagnetic *M-H (x* = 0.1–0.7) are measured at room temperature under an AC magnetic field ~2.27 Oe at 2 kHz, as shown in Fig. 7. The ME coefficients for *x* = 0.1–0.5 gradually increase with the increasing of the applied DC magnetic field, while the ME coefficient for *x* > 0.5 has an opposite behavior, as indicated by the arrows. This difference may be attributed to the different response of the magnetic and electric eigenmodes to the AC magnetic field frequency^48^,^49^. The largest ME coefficient at room temperature is 1.24 mV/cm∙Oe for *x* = 0.5 sample at 4 kOe, comparable with that in 3-layered Bi_4-*x*_Sm_*x*_Ti_3-*x*_Ni_*x*_O_12 ± δ_ (0.6 mV/cm∙Oe)^50^, 5-layered SrBi_5_Fe_0.5_Co_0.5_Ti_5_O_18_ ceramic (0.27 mV/cm∙Oe at room temperature)^8^, cation doped BiFeO_3_ (0.3–2.3 mV/cm∙Oe)^51^, and core-shell 50%CoFe_2_O_4_-50%BaTiO_3_ (3.4 mV/cm∙Oe)^52^. Besides, as mentioned above, some Fe/Co-rich spinel inclusions were observed in the samples. This would lead to the formation of a 0–3 type multiferroic composite, in which another kind of ME coupling effect induced by the magnetostrictive effect from the magnetic phase and the piezoelectric effect from the piezoelectric phase could be obtained^53^. For example, in *x*CoFe_2_O_4_-(1-*x*)Bi_4_Ti_3_O_12_ composite, a much smaller ME coefficient about 0.16 mV/cm∙Oe is observed for *x* = 0.6^54^. However, considering the maximal volume fractions of the magnetic inclusions in our samples are smaller than 0.09%, the contribution to the ME coefficient from the inclusions can be neglected^53^.

## Conclusions

In summary, the 4- and 3-layered intergrowth Aurivillius ceramics Bi_4_NdTi_3_Fe_1-*x*_Co_*x*_O_15_-Bi_3_NdTi_2_Fe_1-*x*_Co_*x*_O_12-*δ*_ with a natural superlattice structure were successfully synthesized, offering us a platform to investigate the relationship between the superlattice structure and physical properties *via* conventional methods. By increasing the cobalt doping concentration, the 4-layered parent phase gradually transforms to 3-layered phase, corresponding to a structure evolution. The 4-layered and 3-layered phase can clamp or modulate each other *via* the lattice mismatch. So the observed properties are beyond a simple combined effect of the two phases. As cobalt doping level increases, the ferromagnetism appears in the ferroelectric material, and the remanent magnetization gradually increases and reaches the maximum value at *x* = 0.3, accompanied by the decreasing of remanent ferroelectric polarization. When further increasing the cobalt doping concentration, the remanent magnetization decreases along with the increasing of ferroelectricity. The variation of magnetic and ferroelectric properties can be well explained by the superlattice structure evolution. Furthermore, all samples with ferromagnetic *M-H (x* = 0.1–0.7) show ME effect at room temperature, and the largest ME coefficient is in *x* = 0.5 sample.

## Methods

The polycrystalline intergrowth superlattice structure Bi_4_NdTi_3_Fe_1-*x*_Co_*x*_O_15_-Bi_3_NdTi_2_Fe_1-*x*_Co_*x*_O_12-*δ*_ (BNTFC-*x*) with *x* = 0, 0.1, 0.3, 0.5, 0.7, 0.9 and 1.0 were prepared by a conventional solid-state reaction method. Note that all the samples were synthesized with 4-layered nominal composition as Bi_4_NdTi_3_Fe_1-*x*_Co_*x*_O_15_. The stoichiometric amounts Bi_2_O_3_ (with 10 wt. % excess Bi_2_O_3_ to compensate volatilization loss during the sintering process), Nd_2_O_3_, Fe_2_O_3_, Co_2_O_3_, and TiO_2_ powders were mixed by grinding. The mixtures were then pre-sintered at 850 °C for 20 h, and subsequently grounded, pelletized and calcined at 900 °C for 20 h. The obtained samples were cut into the form of pellets with the area of 4 × 4 mm^2^. For electrical measurement, the samples were well-polished to thickness of 0.120 mm, and deposited Au electrodes onto the opposite surfaces by sputtering. Due to the extra amount of Bi and the generation of secondary phase, the stoichiometries discussed are nominal (see Supplementary information Table S2 for actual stoichiometry determined by EDS).

Crystalline structures of the samples were characterized by powder X-ray diffraction (XRD) using Cu *K*_*α1*_ radiation (Philips X’Pert Pro diffractometer), and high-angle annular dark-field (HAADF) images (JEOL JEM-2010 field emission electron microscope). Ferroelectric measurement was performed on Radiant Technologies Precision Premier II (Radiant Tech., USA). Magnetic properties were measured using a SQUID-VSM (Quantum Design, USA). The scanning electron microscopy (SEM) images (including secondary-electron (SE) and energy selective backscatter (ESB) images) and EDX were performed on Zeiss Gemini SEM 500 equipped with an ESB detector and an Oxford X-Max 80 detector. The magnetoelectric (ME) voltage coefficient was determined by measuring the electric field generated across the sample with ac magnetic fields (*H*_*ac*_ about 2 Oe) and *dc* bias fields (*H*_*dc*_ up to 5 kOe), performed on Super M-E system (Quantum Design). A signal generator amplified by a power amplifier was used to drive a Helmholtz coil to generate the small *H*_*ac*_ superimposed on *H*_*dc*_. The voltage generated across the sample was measured with a lock-in amplifier. The ME measurement was performed at room temperature.

## Additional Information

**How to cite this article**: Zhang, D. L. *et al*. Structure Evolution and Multiferroic Properties in Cobalt Doped Bi_4_NdTi_3_Fe_1-*x*_Co*_x_*O_15_-Bi_3_NdTi_2_Fe_1-x_Co*_x_*O_12-*δ*_ Intergrowth Aurivillius Compounds. *Sci. Rep.* **7**, 43540; doi: 10.1038/srep43540 (2017).

**Publisher's note:** Springer Nature remains neutral with regard to jurisdictional claims in published maps and institutional affiliations.

## Supplementary Material

Supplementary Material
