# Supplementary material for: Structure Evolution and Multiferroic Properties in Cobalt Doped Bi4NdTi3Fe1-xCoxO15-Bi3NdTi2Fe1-xCoxO12-δ Intergrowth Aurivillius Compounds
Source: Sci Rep. 2017 Mar 8;7:43540. doi: 10.1038/srep43540 (PMC5341086; doi:10.1038/srep43540)
Supplement: Supplementary Material [file srep43540-s1.pdf]

Supplementary material

Structure Evolution and Multiferroic Properties in Cobalt Doped $Bi_4NdTi_3Fe_{1-x}Co_xO_{15}$-$Bi_3NdTi_2Fe_{1-x}Co_xO_{12-\delta}$ Intergrowth Aurivillius Compounds

D. L. Zhang,[1] W. C. Huang,[1] Z. W Chen,[1] W. B. Zhao,[1] L. Feng,[1] M. Li,[1] Y. W. Yin,[1, 2, a)] S. N. Dong,[1] X. G. Li[1, 3, 4, a)]

[1]*Hefei National Laboratory for Physical Sciences at the Microscale, Department of Physics, University of Science and Technology of China, Hefei 230026, China*

[2]*Department of Physics and Astronomy, University of Nebraska, Lincoln, NE 68588, USA*

[3]*Key Laboratory of Materials Physics, Institute of Solid State Physics, CAS, Hefei 230026, China*

[4]*Collaborative Innovation Center of Advanced Microstructures, Nanjing 210093, China*

I. Statistical analysis of second phase magnetic inclusions: volume fractions and magnetic contributions

In previous researches, M. Palizdar *et al.*,[1] L. Keeney *et al.*[2] and M. Schmidt *et al.*[3] provided an original methodology for the detection, localization and quantification of second phase inclusions in Aurivillius phase *via* energy selective backscatter (ESB) image and energy dispersive X-ray analysis (EDX). The backscatter electron is sensitive to the mean atomic number,[4] therefore it is one of the ideal techniques for searching for possible secondary phases. In an ESB image, high atomic number regions appear bright, while low atomic number regions appear dark.[1] To determine the magnetic contribution from the ferromagnetic secondary inclusions, the scanning electron microscopy (SEM) images (including secondary-electron (SE) images and ESB images) and EDX were performed for all $Bi_4NdTi_3Fe_{1-x}Co_xO_{15}$-$Bi_3NdTi_2Fe_{1-x}Co_xO_{12-\delta}$ (BNTFC-*x*, *x* = 0.0, 0.1, 0.3, 0.5, 0.7, 0.9, and 1.0) samples on Zeiss Gemini SEM 500 equipped with an ESB detector and an Oxford X-Max 80 detector.

Taking *x* = 0.3 sample as an example, Fig. S1 shows the ESB image and corresponding Bi, Co and Fe intensity maps (acquired from the EDX mapping). The circled region (with black contrast in Fig. S1 (a)) are Co and Fe rich (Bi poor) areas,

[a)] Author to whom correspondence should be addressed. Electronic mail: lixg@ustc.edu.cn and yyin11@unl.edu

demonstrating the existence and location of the Fe and Co-rich secondary phase.[1]

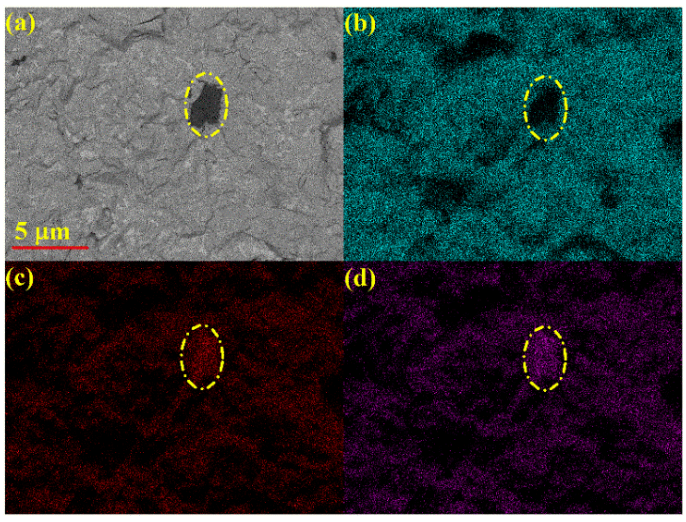

Fig. S1. (a) ESB image and corresponding (b) Bi, (c) Fe and (d) Co intensity map for x = 0.3 sample. The inclusions are marked by a circle.

Figs. S2 (a) and (b) show the SE image and corresponding ESB image of $x = 0$ sample on a relatively large area (10^4 μm^2). Comparing the two images, all dark regions in the ESB image of Fig. S2 (b) are related to the pores or grain boundaries. The ESB image together with the EDX mapping was acquired on a smaller area (25 μm^2) to further verify whether there exist Fe-rich regions, as shown in Figs. S2 (c) and (d). The Fe K intensity map shows no obvious Fe-rich region, probably indicating that no Fe-rich secondary phase was formed in $x = 0$ sample.

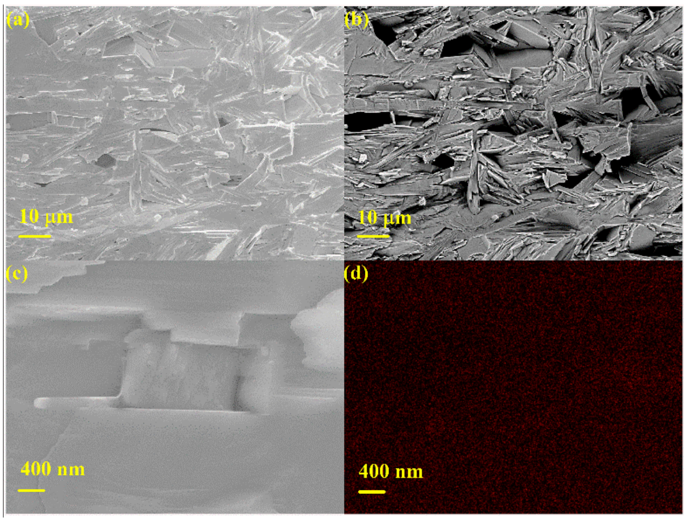

Fig. S2. (a) SE image and (b) corresponding ESB image of $x = 0$ sample on an area of 10^4 μm^2; (c) ESB image and (d) Fe K intensity map for $x = 0$ on an area of 25 μm^2.

For x = 0.1 - 1.0, series measurements with different scanning area sizes and different minimal detectable grain dimensions, were made to estimate the volume fraction of the inclusions, as shown in Figs. S3-S8.The mean atomic number Z_m of the three phases, $Bi_{12}TiO_{20}$, BNTFC-x, and $Fe_{3-y}Co_yO_4$ ($0 \le y \le 3$), have a relationship of Z_m=35.7 ($Bi_{12}TiO_{20}$) > 25.1-25.5 (NTFC-x) > 15.7-16.1 ($Fe_{3-y}Co_yO_4$), and therefore in ESB images, $Bi_{12}TiO_{20}$ phase should be brighter and $Fe_{3-y}Co_yO_4$ inclusions should be darker. Take x=0.5 in Fig. S5 for example, the black regions (such as the black area marked by a red circle) represent the Fe/Co rich spinel magnetic inclusion phase. While the gray regions, which have the largest area, denote the main phase. In addition, as discussed in the main text, with increasing the Co doping level x, an impurity phase $Bi_{12}TiO_{20}$ with higher Bi composition appears, which is the brightest in the ESB images (*e.g.* the white area marked by a blue circle). These are also supported by the local EDS measurements (see following discussion). For x = 0.1 - 1.0, representative areas for the magnetic inclusions (black in ESB image) and $Bi_{12}TiO_{20}$ phase (white in ESB image) are marked out representatively by red and blue circles in Figs. S3-S8.

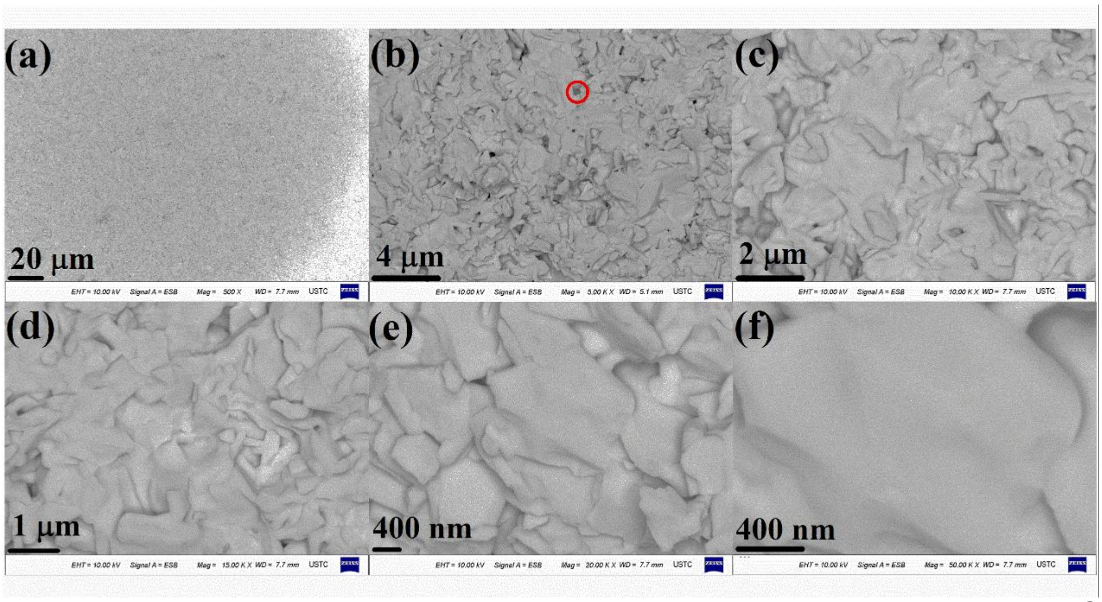

Fig. S3. ESB images for x = 0.1 sample with different scan area sizes, (a) 40000 μm^2, (b) 400 μm^2, (c) 100 μm^2, (d) 44.4 μm^2, (e) 25 μm^2, and (f) 4 μm^2. The bright circle at the corner of (a) is due to the 500× magnification factor approach the limit of the reception range of the ESB detector. A black inclusion in (b) marked by a red circle is a representative Fe/Co rich spinel phase.

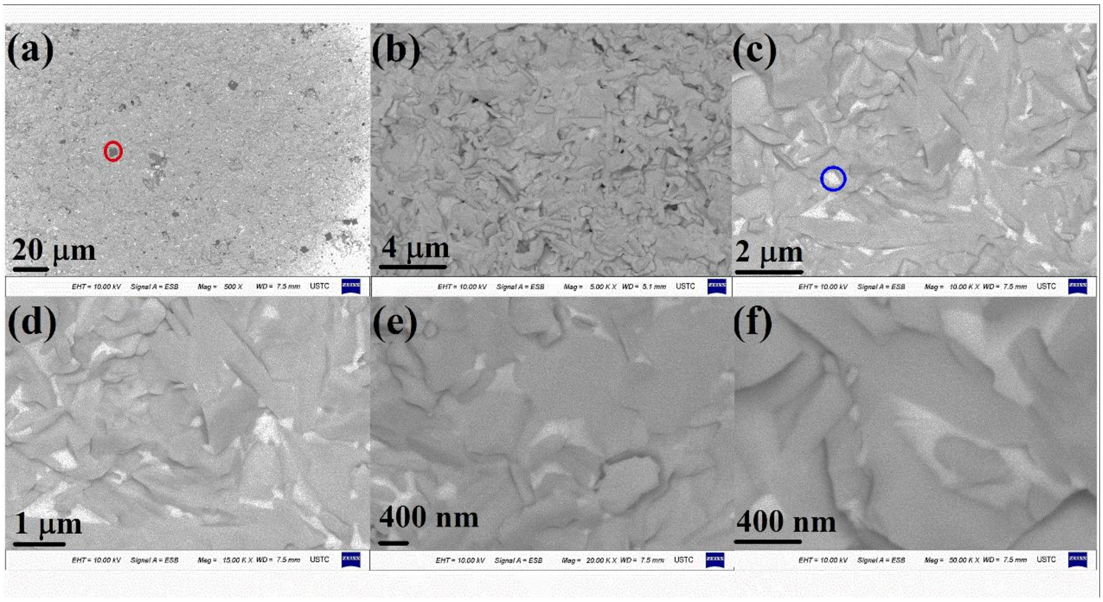

Fig. S4. ESB images for x = 0.3 sample in different magnification factors, (a) 40000 μm^2, (b) 400 μm^2, (c) 100 μm^2, (d) 44.4 μm^2, (e) 25 μm^2, and (f) 4 μm^2. A black inclusion in (a) marked by a red circle is a representative Fe/Co rich spinel phase, and a white one in (c) marked by a blue circle is $Bi_{12}TiO_{20}$

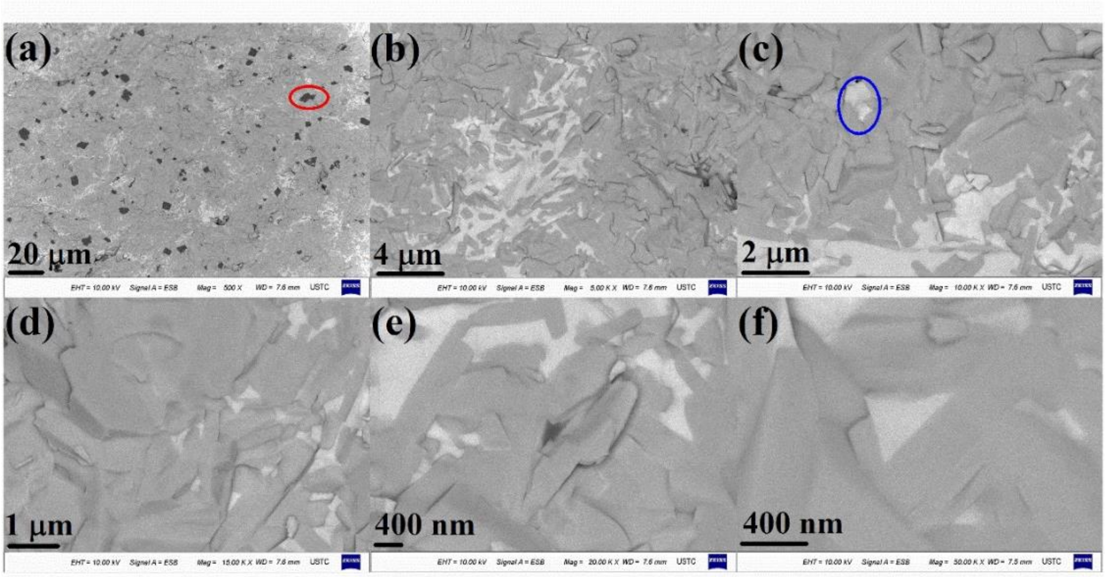

Fig. S5. ESB images for x = 0.5 sample in different magnification factors, (a) 40000 μm^2, (b) 400 μm^2, (c) 100 μm^2, (d) 44.4 μm^2, (e) 25 μm^2, and (f) 4 μm^2. A black inclusion in (a) marked by a red circle is Fe/Co rich spinel phase, and a white one in (c) marked by a blue circle is $Bi_{12}TiO_{20}$.

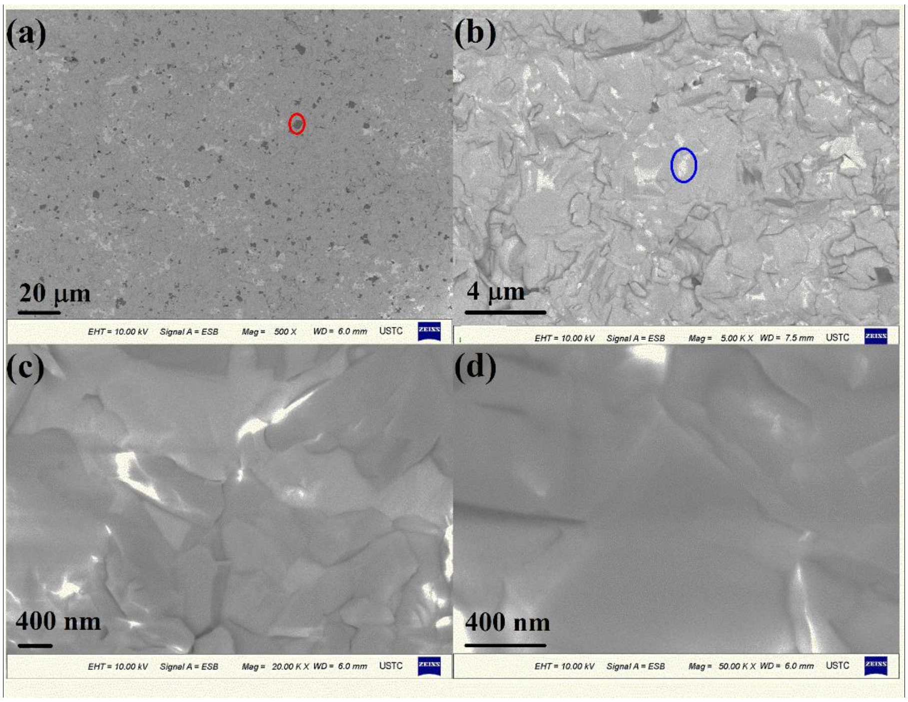

Fig. S6. ESB images for $x = 0.7$ sample in different magnification factors, (a) 40000 μm^2, (b) 400 μm^2, (c) 25 μm^2, and (d) 4 μm^2. A black inclusion in (a) marked by a red circle is Fe/Co rich spinel phase, and a white one in (b) marked by a blue circle is $Bi_{12}TiO_{20}$.

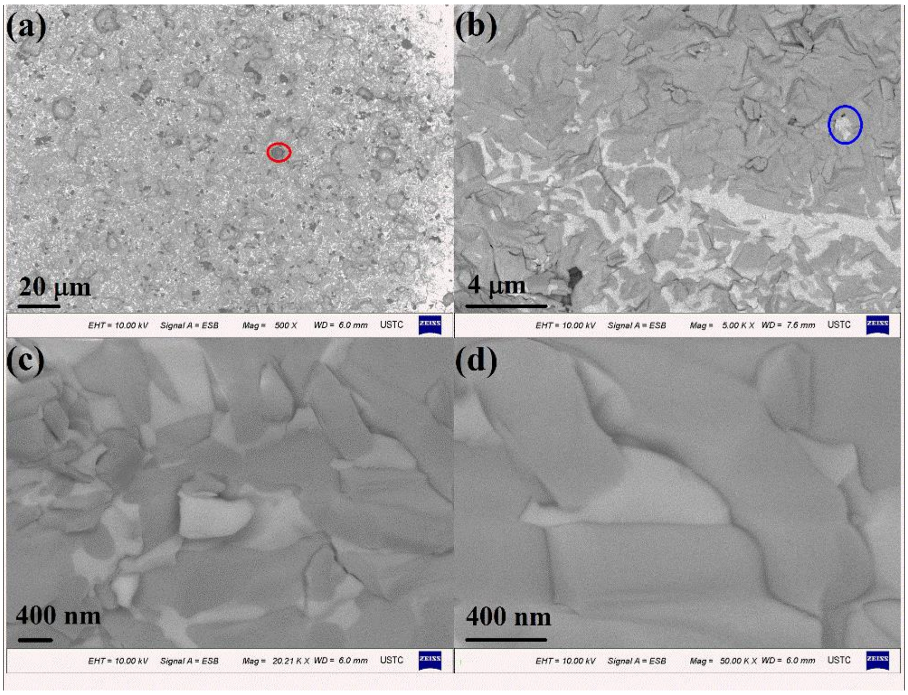

Fig. S7. ESB images for $x = 0.9$ sample in different magnification factors, (a) 40000 μm^2, (b) 400 μm^2, (c) 25 μm^2, and (d) 4 μm^2. A black inclusion in (a) marked by a red circle is Fe/Co rich spinel phase, and a white one in (b) marked by a blue circle is $Bi_{12}TiO_{20}$.

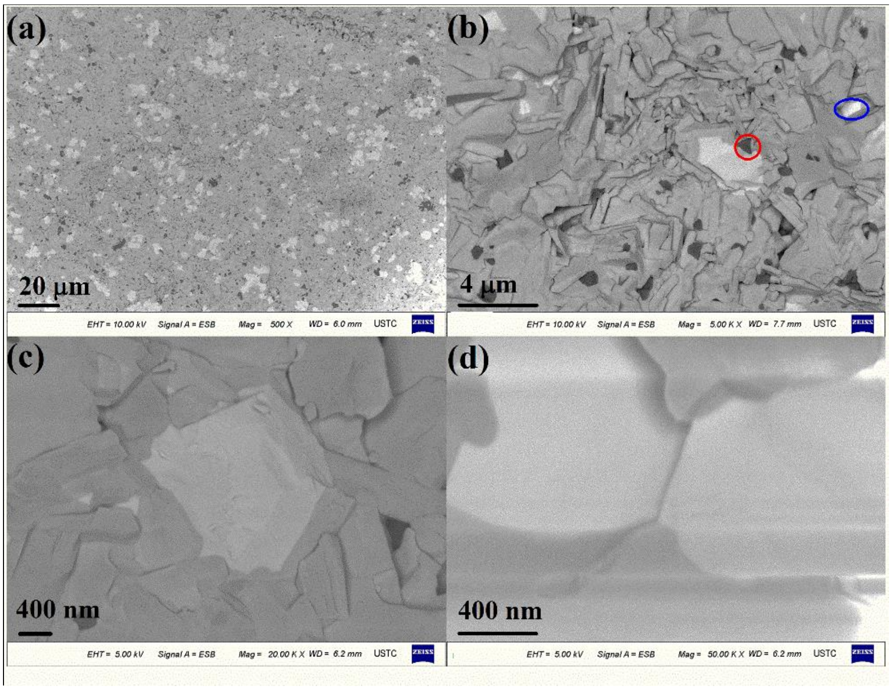

Fig. S8. ESB images for x = 1.0 sample in different magnification factors, (a) 40000 μm^2, (b) 400 μm^2, (c) 25 μm^2, (d) and 4 μm^2. A black inclusion in (b) marked by a red circle is Fe/Co rich spinel phase, and a white one marked by a blue circle is $Bi_{12}TiO_{20}$.

Generally, the Fe and Co-rich magnetic inclusions have a chemical formula $Fe_{3-y}Co_yO_4$ ($0 \leq y \leq 3$), and its remanent magnetization at room temperature decreases as Co content increases.[5-10] In order to give a reasonable estimation of the magnetic contribution from the inclusions, the chemical compositions of the inclusions for all samples were calculated from the EDX mapping. The mole ratios of Fe/Co (Δ) in the inclusions can be obtained by analyzing the inclusion intensities of the EDX mapping.

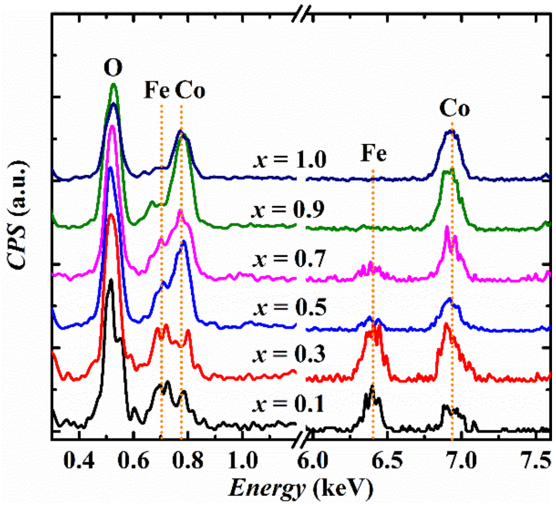

Fig. S9. Energy dispersive X-ray spectrometry (EDS) of the inclusions for x = 0.1-1.0 samples.

Fig. S9 shows the EDS (energy dispersive X-ray spectrometry) of the inclusions for x = 0.1-1.0 samples. It can be clearly seen that the intensity ratio of Fe/Co gradually decreases with increasing Co concentration. Based on the chemical formula of the magnetic inclusions $Fe_{3-y}Co_yO_4$ ($0 \le y \le 3$)[3,5-10], the chemical compositions can be calculated by $y = 3/(1+\Delta)$. For x = 0.1 and 0.3, the Fe/Co mole ratios (Δ) of the inclusions are determined to be 1.17±0.25 and 0.91±0.24, respectively. Thus the inclusions would be $Fe_{1.61}Co_{1.39}O_4$ and $Fe_{1.43}Co_{1.57}O_4$ respectively, and M_{Ir} (the remanent magnetization of the inclusions) could be set to 17 emu/g (the worst case scenario is Fe_2CoO_4 (13-17 emu/g)[7]). For x = 0.5, the Fe/Co mole ratio of the inclusions is 0.37±0.18, indicating the inclusions may be Co-rich spinel phases $Fe_{0.8}Co_{2.2}O_4$ having M_{Ir} of 5.5 emu/g.[10] For x = 0.7, the Fe/Co mole ratio of the inclusions is 0.17±0.12, and the chemical formula for the inclusions would be $Fe_{0.44}Co_{2.56}O_4$ with M_{Ir} of 0.73 emu/g ($Fe_{0.5}Co_{2.5}O_4$).[10] For x = 0.9 and 1.0, the Fe/Co mole ratios of the inclusions are determined to be 0. Thus the inclusions should be Co_3O_4, which is paramagnetic at 300K,[10,11] and the M_{Ir} could be set to 0 emu/g at 300K. Besides, the Bi/Ti mole ratio of the white region calculated from a point EDS (Fig. S10) is about 11.7/1, confirming that the white regions represent $Bi_{12}TiO_{20}$ phase.

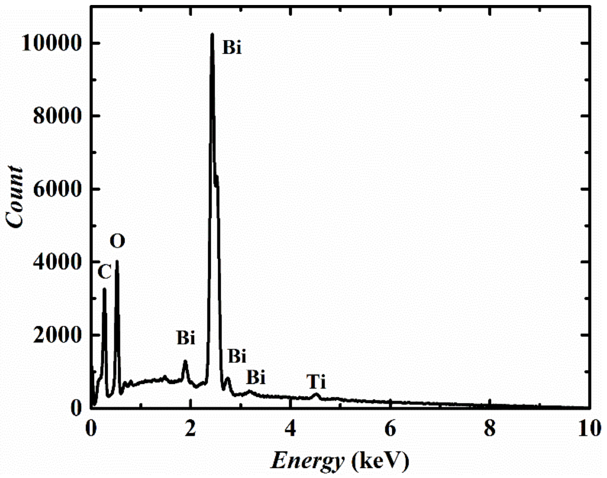

Fig. S10. Energy dispersive X-ray spectrometry (EDS) preformed focus on a white area.

In addition, it is also worth mentioning that the ESB images were acquired at a set resolution of 1024×768 pixels. If the smallest detectable size of inclusions across all scanned areas is defined as 2×2 pixels, which can be distinguished easily, the minimal detectable diameters for scanning areas of 40000 μm^2 (largest scan area) and 4 μm^2 (smallest scan area) are 450 nm and 4.5 nm, respectively. The ferromagnetic

contributions from Fe_3O_4[5,12] and $CoFe_2O_4$[13] inclusions with grain sizes smaller than 5 nm, having a blocking temperature below 300 K, could be neglected at 300 K. In our case, no Fe/Co rich area with a size smaller than 200 nm was observed for all samples. This may be attributed to the high synthesis temperature and long sintering time (pre-sintered at 850 °C for 20 h and calcined at 900 °C for 20 h), which would make the grain size of inclusions grow large.[14]

Assuming that all inclusions are spherical, and following the statistical method proposed by M. Schmidt *et al.*[3], the upper limit of volume fraction $f_{k,u}$ for the inclusions with the sizes between d_k and d_{k-1} ($d_{k-1}>d_k$) can be calculated by

$$f_{k,u} \approx 2.8 \frac{d_{k-1}^3}{\sum_{j=K}^{k} V_j} \tag{1}$$

with a confidence of $\gamma = 99.5\%$, and V_j is an individual scan of a series scans ($k = 1, \ldots, K$).

In order to apply the statistical method to the ESB or EDX images of our samples, formula (1) can be modified to 2-dimension model as follow:

$$f_{k,u,s} \approx 4.16 \frac{d_{k-1}^2}{\sum_{j=K}^{k} S_j} \tag{2}$$

In an isotropic ceramic system, it is reasonable to assume that the inclusions evenly distribute in the samples, thus the volume fraction f_V of the inclusions can be obtained from f_s by

$$f_V = (\sqrt{f_s})^3 \tag{3}$$

Therefore, the volume fraction of the inclusions can be calculated from the ESB images by using equations (1)-(3). Although the size of the inclusions in our samples is larger than 200 nm, the scans with minimal detectable diameter smaller than 200 nm were also performed for statistical analysis. The detailed results are listed in Table S1.

Given the volume fraction of the ferromagnetic inclusions, the maximal contribution from the inclusions (M_i) with diameter between [d_k, d_{k-1}] can be calculated by:

$$M_{i,k} = f_{m,k} M_{Ir,k} = \frac{f_{V,k}\rho_i}{f_{V,k}\rho_i + f_{V,main}\rho_{main} + f_{V,\mathrm{sillenite}}\rho_{\mathrm{sillenite}}} M_{Ir,k} \tag{4}$$

where $M_{Ir,k}$ is the remanent magnetization of the inclusions, $f_{m,k}$ is the mass fraction of the inclusions, $f_{V,main}$ and ρ_{main} are the volume fraction and density of the main phase respectively, $f_{V,sillenite}$ and $\rho_{\mathrm{sillenite}}$ are the volume fraction and density of sillenite

$Bi_{12}TiO_{20}$ respectively. Thus the magnetization remanence from the inclusions has an up bound

$$M_i = \max_{k=1...K}\{M_{i,k}\} \tag{5}$$

with a confidence γ = 99.5%.

Table S1. Performed scans for x = 0.1-1.0 samples. M_r is the remanent magnetization of x = 0.1-1.0 samples, and d_0 is the maximum size of inclusions found in the sample.

x	M_r (memu/g)	Fe/Co ratio	k	Area (μm²)	d_k (nm)	f_V (%)	M_i (memu/g)
0.1	17.3	1.17 (±0.25)	1	40000	450 (d_0=2.86μm)	2.4E-3	0.29
			2	400	45	5.6E-3	0.67
			3	100	22.5	3.4E-5	0.004
			4	44.4	15	1.5E-5	0.002
			5	25	11.25	1.8E-5	0.002
			6	4	4.5	1.5E-4	0.018
0.3	123	0.91 (±0.24)	1	40000	450 (d_0=5.71μm)	1.9E-2	2.31
			2	400	45	5.6E-3	0.67
			3	100	22.5	3.4E-5	0.004
			4	44.4	15	1.5E-5	0.002
			5	25	11.25	1.8E-5	0.002
			6	4	4.5	1.5E-4	0.018
0.5	89.9	0.37 (±0.18)	1	40000	450 (d_0=9.5μm)	9.0E-2	3.31
			2	400	45	5.6E-3	0.21
			3	100	22.5	3.4E-5	0.0012
			4	44.4	15	1.5E-5	0.0006
			5	25	11.25	1.8E-5	0.0007
			6	4	4.5	1.5E-4	0.006
0.7	7.02	0.17 (±0.12)	1	40000	450 (d_0=5.71μm)	2.0E-2	0.11
			2	400	45	8.7E-3	0.05
			3	25	11.25	5.0E-4	0.0026
			4	4	4.5	1.5E-4	0.0008
0.9	0.026	0	1	40000	450 (d_0=6.67μm)	3.1E-2	0
			2	400	45	8.7E-3	0
			3	25	11.25	5.0E-4	0
			4	4	4.5	1.5E-4	0
1.0	0.022	0	1	40000	450 (d_0=7.62μm)	4.6E-2	0
			2	400	45	8.7E-3	0
			3	25	11.25	5.0E-4	0
			4	4	4.5	1.5E-4	0

According to the above analysis, the upper limit impact on magnetic contributions to M_r from the inclusions (M_i) for x = 0.1, 0.3, 0.5, 0.7, 0.9 and 1.0 are 0.67 memu/g, 2.31 memu/g, 3.31 memu/g, 0.11 memu/g, 0 and 0, respectively, as listed in Table S1. It is found that the magnetic contributions of the inclusions are much smaller than the total magnetizations measured for all samples. For the samples with ferromagnetic signals, namely, x = 0.1, 0.3, 0.5, and 0.7, the magnetic contributions to the corresponding main phases of the inclusions are conservatively estimated to be about or smaller than 3.9%, 1.9%, 3.8%, and 1.5%, respectively. Following the criteria of the comprehensive framework raised by M. Schmidt *et al.*[3], this indicates that the magnetic results do represent intrinsic information of the main phase.

II. Actual stoichiometry from EDX analysis for $Bi_4NdTi_3Fe_{1-x}Co_xO_{15}$-$Bi_3NdTi_2Fe_{1-x}Co_xO_{12-\delta}$ (x = 0.0, 0.1, 0.3, 0.5, 0.7, 0.9, and 1.0) samples

The actual stoichiometries (mole ratio, normalized by Ti element) of the samples determined by energy dispersive X-ray analysis (EDX) mapping are listed in Table S2. The mole ratios of Bi, Nd and Ti change little, while those of Fe, Co vary with the nominal doping level. The volume fractions for 4- and 3-layered phases and $Bi_{12}TiO_{20}$ listed in Table S2 are modified by calculating the franction of magnetic inclusions, thus these values have a small difference with those obtained by XRD refinements.

Table S2. The volume fractions and chemical constituents of the samples (normalized by Ti element)

x	Volume Fraction (%)				Bi	Nd	Ti	Fe	Co
	4-layer	3-layer	$Bi_{12}TiO_{20}$	Inclusions*					
0	100	0	0	0	1.35	0.28	1	0.30	-
0.1	99.99	0	0	5.6E-3	1.26	0.37	1	0.28	0.05
0.3	80.09	17.15	2.74	1.9E-2	1.20	0.28	1	0.20	0.10
0.5	42.71	41.71	15.49	9.0E-2	1.20	0.26	1	0.15	0.19
0.7	8.87	77.41	13.70	2.0E-2	1.25	0.28	1	0.08	0.21
0.9	0.29	83.07	16.60	3.1E-2	1.35	0.26	1	0.03	0.27
1.0	0	85.30	14.65	4.6E-2	1.23	0.29	1	-	0.31

* Data from Table S1.

References:

1 Palizdar, M. *et al.* Crystallographic and magnetic identification of secondary phase in orientated $Bi_5Fe_{0.5}Co_{0.5}Ti_3O_{15}$ ceramics. *J. Appl. Phys.* **112**, 073919 (2012).

2 Keeney, L. *et al.* Magnetic Field-Induced Ferroelectric Switching in Multiferroic Aurivillius Phase Thin Films at Room Temperature. *J. Am. Ceram. Soc.* **96**, 2339-2357 (2013).

3 Schmidt, M. *et al.* Absence of evidence not equal evidence of absence: statistical analysis of inclusions in multiferroic thin films. *Sci. Rep.* **4**, 5712 (2014).

4 Itakura, M., Kuwano, N., Sato, K. & Tachibana, S. Variations in contrast of scanning electron microscope images for microstructure analysis of Si-based semiconductor materials. *J Electron Microsc (Tokyo)* **59 Suppl 1**, S165-173 (2010).

5 Goya, G. F., Berquó, T. S., Fonseca, F. C. & Morales, M. P. Static and dynamic magnetic properties of spherical magnetite nanoparticles. *J. Appl. Phys.* **94**, 3520 (2003).

6 Guan, N., Wang, Y., Sun, D. & Xu, J. A simple one-pot synthesis of single-crystalline magnetite hollow spheres from a single iron precursor. *Nanotechnology* **20**, 105603 (2009).

7 El-Dek, S. I. Effect of annealing temperature on the magnetic properties of $CoFe_2O_4$ nanoparticles. *Philosophical Magazine Letters* **90**, 233-240 (2010).

8 Muthuselvam, I. P. & Bhowmik, R. N. Structural phase stability and magnetism in Co_2FeO_4 spinel oxide. *Solid State Sciences* **11**, 719-725 (2009).

9 Panda, M. R., Bhowmik, R. N., Singh, H., Singh, M. N. & Sinha, A. K. Air annealing effects on lattice structure, charge state distribution of cations, and room temperature ferrimagnetism in the ferrite composition $Co_{2.25}Fe_{0.75}O_4$. *Materials Research Express* **2**, 036101 (2015).

10 Kim, K. J., Lee, J. H. & Kim, C. S. Phase decomposition and related structural and magnetic properties of iron-cobaltite thin films. *Journal of the Korean Physical Society* **61**, 1274-1278 (2012).

11 He, L., Chen, C., Wang, N., Zhou, W. & Guo, L. Finite size effect on Néel temperature with Co_3O_4 nanoparticles. *J. Appl. Phys.* **102**, 103911 (2007).

12 Roca, A. G., Morales, M. P., O'Grady, K. & Serna, C. J. Structural and magnetic properties of uniform magnetite nanoparticles prepared by high temperature decomposition of organic precursors. *Nanotechnology* **17**, 2783-2788 (2006).

13 Liu, C., Zou, B., Rondinone, A. J. & Zhang, Z. J. Chemical Control of Superparamagnetic Properties of Magnesium and Cobalt Spinel Ferrite Nanoparticles through Atomic Level Magnetic Couplings. *J. Am. Chem. Soc.* **122**, 6263-6267 (2000).

14 Sedlacik, M., Pavlinek, V., Peer, P. & Filip, P. Tailoring the magnetic properties and magnetorheological behavior of spinel nanocrystalline cobalt ferrite by varying annealing temperature. *Dalton Trans* **43**, 6919-6924 (2014).
